# Genetic and Management Effects on Barley Yield and Phenology in the Mediterranean Basin

**DOI:** 10.3389/fpls.2021.655406

**Published:** 2021-04-15

**Authors:** Davide Cammarano, Domenico Ronga, Enrico Francia, Taner Akar, Adnan Al-Yassin, Abdelkader Benbelkacem, Stefania Grando, Ignacio Romagosa, Antonio Michele Stanca, Nicola Pecchioni

**Affiliations:** ^1^Department of Agronomy, Purdue University, West Lafayette, IN, United States; ^2^Department of Life Science, Centre BIOGEST-SITEIA, University of Modena and Reggio Emilia, Reggio Emilia, Italy; ^3^Department of Pharmacy, University of Salerno, Fisciano, Italy; ^4^Department of Agronomy, Faculty of Agriculture, Akdeniz University, Antalya, Turkey; ^5^National Agricultural Research Center (NCARE), Amman, Jordan; ^6^National Agronomic Research Institute of Algeria (INRAA), Constantine, Algeria; ^7^International Consultant, Perugia, Italy; ^8^Agrotecnio Centre, Universitat de Lleida, Lleida, Spain; ^9^Research Centre for Cereal and Industrial Crops, CREA – Council for Agricultural Research and Economics, Foggia, Italy

**Keywords:** crop model, barley, genotype, management, Mediterranean

## Abstract

Heading time in barley is considered a key developmental stage controlling adaptation to the environment and it affects grain yield; with the combination of agronomy (planting dates) and genetics being some of the determinants of adaptation to environmental conditions in order to escape late frost, heat, and terminal drought stresses. The objectives of this study are (i) to apply a gene-based characterization of 118 barley doubled haploid recombinants for vernalization, photoperiod, and earliness per se; (ii) use such information to quantify the optimal combination of genotype/sowing date that escapes extreme weather events; and (iii) how water and nitrogen management impact on grain yield. The doubled haploid barley genotypes with different allelic combinations for vernalization, photoperiod, and earliness per se were grown in eight locations across the Mediterranean basin. This information was linked with the crop growth model parameters. The photoperiod and earliness per se alleles modify the length of the phenological cycle, and this is more evident in combination with the recessive allele of the vernalization gene VRN-H2. In hot environments such as Algeria, Syria, and Jordan, early sowing dates (October 30 and December15) would be chosen to minimize the risk of exposing barley to heat stress. To maintain higher yields in the Mediterranean basin, barley breeding activities should focus on allelic combinations that have recessive VRN-H2 and EPS2 genes, since the risk of cold stress is much lower than the one represented by heat stress.

## Introduction

Barley is one of the most important cereal crops cultivated in the Mediterranean basin, and thanks to its short growth cycle, it is grown worldwide from the Arctic Circle to the equator, at different elevations (Ceccarelli et al., 2011; Dawson et al., 2015). Mediterranean environments are characterized by a high inter-annual variability of temperature and rainfall patterns which increase the uncertainty of keeping production at greater levels (Cammarano et al., 2019a). Air temperature impacts crop phenology with higher values that accelerate developmental stages, causing a shortening of the growing season and a reduction in grain yield (Francia et al., 2011). In particular, maximum air temperature causes acceleration of leaf senescence rates and a negative impact on grain filling (Asseng et al., 2011). The amount of rainfall impacts the available soil water content that can be used by the crop. And it dictates both the amount of nitrogen that can be taken up, since soil nitrogen is taken by the crop when there is adequate soil water, and the amount of N lost by leaching (Basso et al., 2011; Cammarano et al., 2019a). Therefore, the adoption of agronomic strategies, like choice of adapted cultivars, planting dates, and fertilizer management, can help farmers to optimize production, while exposing heading dates to period with low risks of heat or frost. This is strategic to reduce the yield gap between the potential and actual yield. Therefore, in addition to agronomic management, a breeding strategy to improve barley genotypes by adjusting their phenology will be a pivotal additional tool to maximize barley yield in a Mediterranean environment affected by climate change (Cammarano et al., 2019a).

The adjustment of developmental time of the different phenological stages is important for improving grain yield and to maintain crop performance under various stress conditions (Francia et al., 2011). This is because they can be adjusted to avoid periods of extreme stress (Zheng et al., 2013). Heading (defined as the head emergence from flag leaf) is considered a key developmental stage as it is associated with adaptation to the environment and a determinant of grain yield, which makes it a target trait in barley (Alqudah and Schnurbusch, 2017). In addition, heading is among the primary determinants of adaptation to environmental conditions in order to escape frost, heat, and terminal drought stresses (Rousset et al., 2011; Zhao et al., 2019). Heading date is influenced by several genes responding to mean air temperature (earliness per se, EPS, or Eam), cold air temperature (vernalization, Vrn), and day length (photoperiod, Ppd) (von Zitzewitz et al., 2005; Francia et al., 2011; Rousset et al., 2011). It has been reported that most of the variation in developmental rates can be explained by vernalization and photoperiod response genes, with significant effects of earliness per se alleles (Francia et al., 2011). In the last few years there has been an increasing understanding of the regulative networks of the genes that regulate the mechanism of the passage between vegetative and reproductive stages (Trevaskis et al., 2007; Brambilla et al., 2017).

The interaction between genetic factors, environment (known as GxE), and the agronomic management (GxExM) makes the integration among these different disciplines for research and application challenging (Bertin et al., 2010). In fact, to take into account the GxE of crop traits, many experiments over several years and sites under different environmental conditions are needed (Francia et al., 2011). This is not often possible and researchers have proposed the use of crop growth models for analyzing the impact of the genotype on different plant processes on several crop species (Yin et al., 2000; Chapman et al., 2003; Reymond et al., 2003; Stewart et al., 2003; White et al., 2008; Zheng et al., 2013).

The interactions between soil-plant-atmosphere-agronomic management can be simulated with crop models. They are a process-based representation of the impact of the environment, agronomic management, and cultivars on crop growth processes, development, and yield (Jones et al., 2003; Keating et al., 2003). In such types of models, the different cultivars can be represented by parameters that reflect the difference in phenology, biomass, and yield. The difference in phenology is usually represented by parameters linked with the sensitivity to vernalization and photoperiod (White et al., 2008; Zheng et al., 2013); therefore, they are linked to the response to air temperature, day length, and their interactions. Earliness per se could also be parameterized in crop growth models by modifying parameters associated to the minimum duration of specific development phases (White et al., 2008). Those parameters are often reported in literature as “genetic coefficients” in order to consider their values as differences between cultivars. They are estimated by using field-collected data on phenology (Baenziger et al., 2004). However, the parameterization of cultivar is a time-consuming process when a large number of genotypes need to be calibrated. This reduces the speed at which genotypes can be included into such models. Barley breeding, along with classic agronomy research, would benefit from the use of models that include gene-based parameterization to predict the GxE interactions (White et al., 2008).

The inclusion of genetic information into crop growth models in order to calibrate those parameters has been proposed in many different forms. For example, specific gene or allelic combinations were used to estimate the parameter values (White et al., 2008; Zheng et al., 2013), to design new ideotypes (Bertin et al., 2010; Rötter et al., 2015; Tao et al., 2017), or to quantify the GxE interactions (Yin et al., 2005).

Once the “genetic coefficients” are parameterized using a gene-based approach, the in-silico genotype can be used to simulate the impacts of different environmental and management conditions. On the common bean, such an approach was used to simulate 30 genotypes by parameterizing the effect of eight genes affecting phenology, growth habit, and seed size (White and Hoogenboom, 1996; Hoogenboom and White, 2003). A similar approach was also proposed and modified to define the effects of six loci on the growth and development of soybean lines (Messina et al., 2006).

The determination of genetic coefficients through gene-based calibration can be achieved in different ways. However, the choice depends on the type of data available because not all the experimental data are suitable for a particular gene-based calibration. On wheat, several approaches have been used to include the effects of genes for photoperiod and vernalization for calibrating the crop growth models. Some approaches used data collected under vernalization and photoperiod treatments and used a one-step optimization to estimate the crop growth model parameters (Zheng et al., 2013). On barley, a linkage between QTL information and a crop growth model was made to predict the yield of recombinant inbred lines (Yin et al., 2000). While these would be good approaches they require specifically designed experiments or datasets. When such types of experiments were not available, multi-locations/cultivars data and a different statistical approach to estimate genetic coefficients was used (White et al., 2008).

In a study about the climate impacts on barley grown in the Mediterranean basin, the impacts of climate variability and projected changes were quantified (Cammarano et al., 2019a). Days with higher maximum air temperature above 34 °C reduced both crop development and soil water content, which results in lower yields even for wetter climate projections (Cammarano et al., 2019a). However, that study was done using a generic barley cultivar calibrated and evaluated at several sites where many genotypes were compared. A characterization of barley genotypes in the Mediterranean basin will improve the understanding on how climate variability and extreme events impact each genotype.

Agronomic management, such as planting date, also has an important impact on adaptation to such types of environments. In fact, early or late sowing times can expose crops to frost, heat, or terminal drought events. The combination of genotypes, having different flowering times, and sowing time will offer a useful insight on the adaptation to local environmental conditions.

Therefore, the objectives of this study are (i) to apply a gene-based characterization of different barley genetic types for vernalization and photoperiod; (ii) use such information to quantify the optimal combination of genotype/sowing date that escapes extreme weather events; and (iii) how water and nitrogen management impact on grain yield.

## Materials and Methods

### Study Areas

**FIGURE 1:**
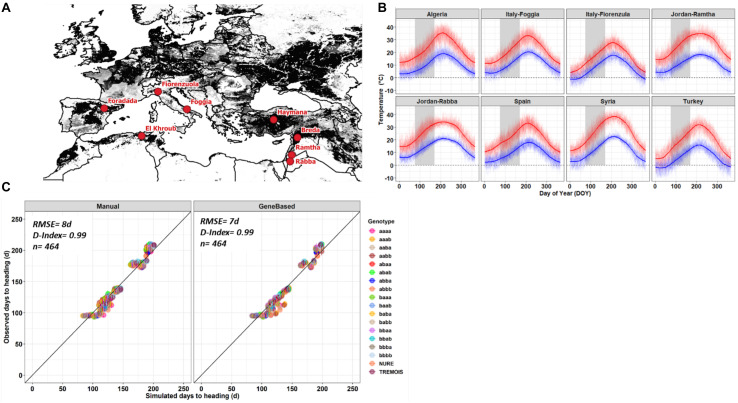
**(A)** Barley growing area in the Mediterranean basin (gray scale area) and the eight locations (red dots), where, the barley genotypes were grown. **(B)** Maximum (red line) and minimum (blue line) air temperature at each location, the gray area represents the heading dates window simulated using long-term weather data. **(C)** Manual (left panel) and gene-based (right panel) fit against observed heading dates for each of the eighteen genetic types. Error bars represent the standard deviation of number of days across the replicates and the lines used for each allelic combination.

Eight locations across the Mediterranean basin were selected where two different studies were published with the same environmental, management, and cultivar information (Figure 1A; Francia et al., 2011; Cammarano et al., 2019a). There was one location in northeastern Spain (Foradada), two locations in Italy (Foggia and Fiorenzuola), one in Algeria (El Khroub), two locations in Jordan (Ramtha and Rabba), one in Syria (Breda), and one in central Turkey (Haymana). In the manuscript, the Figures showing two locations in the same country will be reported; while for the countries with single locations only the country name will be reported.

Crop management in terms of nitrogen and water (where supplementary irrigation was applied), and all the other detailed information is reported elsewhere (Francia et al., 2011; Cammarano et al., 2019a) and the main environmental information is summarized in Supplementary Table 2. Heading and maturity dates were recorded, although not in all the locations (Supplementary Tables 3, 4). Grain yield for each of the hundred and eighteen cultivars was recorded as well.

One hundred and eighteen doubled haploids derived by the cross NURE × TREMOIS (NxT) were grown in each location. The NxT was also known as IBMP (Italian Barley Mapping Population), and was a biparental population used to build a genetic map, to map QTLs for stress tolerance, as well as one of the genetic materials used for cloning VRN-H1/VRN-H2, and EPS2 (Earliness Per Se 2) genes in barley (von Zitzewitz et al., 2005; Francia et al., 2011; Comadran et al., 2012). Nure [(Fior40 x Alpha^2) x Baraka] was a winter, two-rowed, feeding variety released by the Istituto Sperimentale per la Cerealicoltura, Italy in 1998, adapted to South European environments. Tremois [(Dram x Aramir) x Berac] was a spring, two-rowed, malting cultivar released by Verneuil-Limagrain, France in 1989, adapted to fertile environments. The former has high cold tolerance, while the latter is adapted to fertile environments and susceptible to low temperature. The locations had contrasting soil water holding capacities and different growing season conditions. Additional information regarding the details of the experiments is reported in previous works (Francia et al., 2011; Cammarano et al., 2019a). It constitutes an experimental population well suited to study the barley genes that have an impact on adaptation to the environment.

These doubled haploids have been grouped into similar allelic combinations using information about vernalization (VRN-H1, VRN-H2), photoperiod (PPD-H2), and earliness per se (EPS2) genes to cluster them into 16 combinations of homozygous genotypes, with fixed alleles at the three loci. Despite being composed of different ratios of parental alleles in all the remaining genomic regions, including NURE and TREMOIS, carrying the two parental genomes as well as the two sets of phenology genes males a total of 18 allelic combinations to be considered (Supplementary Table 1).

### Climate Data

At each location, one growing season of daily weather data was available. This weather data was used for model calibration and evaluation. But, to simulate the impact of long-term climate on heading dates and grain yield, the NASA-AgMERRA product was used for the 30-year period 1980-2010 (Ruane et al., 2015). Daily values of solar radiation (MJ m^–2^ d^–1^), maximum temperature (°C), minimum temperature (°C), and rainfall (mm) were downloaded from the online NASA-AgMERRA database (Ruane, 2021). This dataset has been used in several studies of climate-related impacts (Cammarano et al., 2019a). A previous study compared the observed weather data to the NASA-AgMERRA finding a good fit between the observed and derived weather parameters (Cammarano et al., 2019a,b).

### Crop Simulation

A crop simulation model (DSSAT v4.7) (Hoogenboom et al., 2019) was used for simulating each allelic combination. The inputs for the model are daily weather data, soil and crop management data, and soil water and nitrogen amount when the model starts the simulation runs (known as initial conditions). Soil, management, and initial conditions are available at each site as a result of a previous modeling work (Cammarano et al., 2019a). The whole dataset was subdivided into a calibration and an evaluation dataset. The data for the calibration were from the irrigated trials that were made in Foggia and Syria (Supplementary Table 2). This is because the calibration of crop parameters is usually done in nearly ideal conditions in terms of water and nutrients (Kersebaum et al., 2015). All the allelic combinations were hand-calibrated by matching observed and simulated heading, maturity, and yield data. The evaluation was done using all the other data available from the other locations for the same observed parameters.

In the DSSAT, the barley model used for this study is the CERES-Barley. The developmental rates are function of air temperature and photoperiod. The CERES-Barley calculates mean daily temperature using air maximum and minimum temperature. When vernalization and photoperiod effects are not present the developmental rates linearly increase for temperatures above 0°C, which is the base temperature, up to the optimum 26°C that is the threshold above which crop development rates advances at a maximum. When the accumulated effective temperature has been reached, then a particular developmental stage is reached (Jones et al., 2003; White et al., 2008). When vernalization and photoperiods are considered, the calculation of the accumulated effective temperature also considers two factors, the vernalization and photoperiod. In the DSSAT-Barley the cultivar-specific parameters for vernalization and photoperiod are called P1V and P1D. The former is defined as the number of days of vernalization that are needed for flowering to occur, with the assumption that temperatures for vernalization are optimal. In fact, vernalization occurs at temperatures from −5 to 15°C with the maximum between 0°C and 7°C (White et al., 2008). The devernalization process is also considered and happens when less than 10 days of progress to vernalization have been accumulated and the maximum temperature is 30°C. The accumulation of daily rates of vernalization is then used to compute the vernalization factor (Jones et al., 2003). The photoperiod parameter within the CERES-Barley (P1D) is the photoperiod response, expressed as percentage reduction in developmental rate in a photoperiod 10 h shorter than the critical long photoperiod. The critical long photoperiod is a threshold above which there is no additional effect of photoperiod on development, which is set to 20 h. Earliness per se was calibrated by modifying the thermal time between the end of the juvenile stage, as defined in Ritchie (1991), and terminal spikelet (which is known as P1 parameter).

After calibration and evaluation we applied a published approach (White et al., 2008), to include the effects of the different alleles for each set of crop parameters. The new set of P1V, P1D, and P1 were estimated by linear models that considered the additive and epistatic effects of VRN-H1 and VRN-H210, the Ppd-H2 and the EPS2 in the form:

(1) $$P\,1\,V=23.4+10.85\,V\,r\,n\,H\,1+1.6\,V\,r\,n\,H\,2+11.75\,V\,r\,n\,H\,1VrnH2\left(r^{2}=0.92,p<0.01\right)$$

(2) $$P1D=76.778+1.778PpdH2\left(r^{2}=0.10,p=0.7\right)$$

(3) $$P1=380-75Eps2\left(r^{2}0.20,p=0.04\right)$$

In this study, we also wanted to quantify a mean yield impact per allelic group; since in each allelic group there were different cultivars, a mean yield and a standard deviation were used to plot the results of calibration and evaluation (Supplementary Figure 2). In this case the dataset was split between some irrigated experiments and cool climate data (Italy-Fiorenzuola and Turkey) that were used for calibrating the mean yield response; and the remaining dataset (including some other irrigated treatments) used for the evaluation (Supplementary Figure 2).

Long-term simulations were set up to run for each growing season from 1980 to 2010. Since we aimed at quantifying the climate impacts, the crop model was re-set with the same set of initial conditions every year. In this way, the only variability within the system comes from the weather data.

In order to take into account the sowing window of barley within the Mediterranean environment, eight sowing dates were used as input in the model, from October 30 until February 15 every 15 days. In addition, four different management strategies were simulated. In the current experimental setup some locations grew barley both under irrigated and rainfed conditions (Francia et al., 2011). Therefore, for each location we decided to consider the impacts of nitrogen and water management as well. The experiments were simulated with no additional water applied (**Dry**); with a deficit-irrigation, applying water when the available soil water content in the first 50 cm of soil fell below 40% and stopped when it reached the 80%. Nitrogen was applied either using the amounts reported in each experiment and reported elsewhere (Francia et al., 2011; Cammarano et al., 2019a) (**Nmgt**). In addition, the model was simulated by not considering any nitrogen stress as a reflection of a potential optimal nitrogen management (**Nopt**). The management combinations to be simulated are: (i) no additional water and nitrogen management reported in a previous study (Francia et al., 2011) (**DryNmg**t); (ii) no additional water and optimal nitrogen management (**DryNopt**); (iii) irrigated and reported nitrogen management (**IrrNmgt**); and (iv) irrigated and optimal N management (**IrrNopt**).

### Data Analysis

The comparison between observed and simulated data for the calibrated and evaluated dataset was done using two different statistics. The Root Mean Square Error (RMSE) was calculated as follows:

(4) $$R\,M\,S\,E=\sqrt{\frac{1}{n}\,\sum_{i=1}{\left(O_{i}-S_{i}\right)}^{2}}$$

where *O_i* are the observations, *S_i* the simulations, and *n* is the number of comparison. The other statistical index used to compare the simulations and observations is the Wilmott index of agreement (D-Index) (Wilmott, 1982). Values of this index range from 0 (poor fit) to 1 (good fit). Such an index is a descriptive measure and is generally used to make cross-comparisons between models (Wilmott, 1982).

(5) $$D-I\,n\,d\,e\,x=1-\frac{\sum_{i=1}^{n}{\left(O_{i}-S_{i}\right)}^{2}}{\sum_{i=1}^{n}{\left(\left|O_{i}-\bar{O}\right|+\left|S_{i}-\bar{O}\right|\right)}^{2}}$$

where $\bar{O}$ is the mean of the observed data. The results of the statistical comparison are reported in Figure 1C and Supplementary Figures 1, 2.

For each growing season and planting date the first day of heat and last day of frost were calculated and the values converted into cumulative probability functions using the same approach reported elsewhere (Zheng et al., 2013). Briefly, the optimal window was identified as the period where the risk is lower than 10% for frost (<0 °C) and a risk lower than 40% for a heat event (>35°C).

The Figures were drawn using the R software base (R Core Team., 2021) and the GGPLOT2 package (Wickham, 2016); while the heat/frost days were determined using the R software (R Core Team., 2021). Figure 1 was drawn in QGIS by modifying the open-source MapSPAM dataset (Yu et al., 2020).

## Results

### Characterization of Barley Genotypes

The 118 doubled haploid lines, being homozygous offspring of a winter x spring, early x late cross, are carrying different combinations of four genes that regulate the plant phenology in response to temperature and day length. As better described in the section “Materials and Methods,” they were grouped into 16 genetic groups each carrying a homozygous combination of the four genes. The different allelic combinations shown in Figure 1 and used to group the barley lines were, in order, VRN-H1, VRN-H2, PPD-H2, and EPS2; after the inclusion of the two parental data, 18 genetic types have been characterized. The 18 barley genetic types, grown experimentally at eight locations, were calibrated in a crop growth model. They showed better fit with the observed data if the inclusion of the four genes was considered (Figure 1). The eight locations are distributed across the Mediterranean basin (Figure 1A) and show distinct environmental conditions in terms of maximum and minimum air temperature (Figure 1B). Three locations, Spain, Italy-Fiorenzuola, and Turkey, being cooler environments than the others, as their minimum air temperature could be below 0°C during winter times (Figure 1B). The manual calibration of the eighteen genotypes showed a good fit between observed and simulated heading date (RMSE = 8 days, D-Index = 0.99, and *n* = 464) (Figure 1C, left panel); on the other hand, the gene-based approach showed a slight lowering of the error and a similar goodness of fit index (RMSE = 7 days, D-Index = 0.99, and *n* = 464) (Figure 1C, right panel). The calibration and evaluation of maturity date and yield were shown in Supplementary Figures 1, 2.

### Planting Time and Alleles Combinations as Strategy for Escaping Late Frost and Heat

**FIGURE 2:**
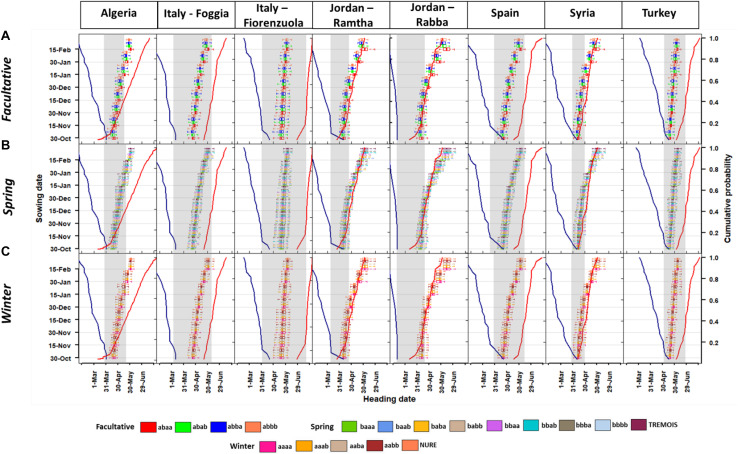
Boxplots of the heading date at different sowing times for the **(A)** facultative, **(B)** spring, and **(C)** winter barley types. The secondary axis shows the probability for the last frost days (blue line on the left) and first heat days (red solid line on the right), these are calculated as the percentile of last frost days (<0°C) and first heat days (>35°C) from 1980 to 2010 and defined as 10% of risk for the last day of frost and 40% for first day of heat as defined in previous work (Zheng et al., 2013). For each boxplot, the end of the horizontal line represents, from the left to the right, the 10th percentile and the 90th percentile. The vertical line of the box, from the left to the right represents the 25th and 75th percentile, respectively.

To test how the different barley genetic types responded to various management strategies, they were divided into facultative, spring, and winter types. The risk of last day of frost and first day of heat at each location is also showed in Figure 2. The optimal heading time window (gray shaded area) at each location indicated that there is, for each allelic combination, an optimal planting date that minimizes the risk of cold/heat on heading dates. This means that in the pseudo-spring facultative genotype, the vernalization requirement has been eliminated. In the spring type, the presence of the dominant VRN-H1 allele confers the spring growth habit regardless of the allelic constitution of VRN-H210,27. In fact the VRN-H2 locus is present in winter genotypes and deleted in facultative and spring genotypes (von Zitzewitz et al., 2005).

Overall, late planting dates at most locations put the genotypes at risk of heat stress (like Algeria, Jordan and Syria), while in other locations (e.g., Italy, Spain, and Turkey) the planting dates are within the safe window (Figure 2). The mean difference between facultative, spring, and winter genotypes was of about 3 days (values averaged across all the genotypes within each group) with the facultative being the earliest and the winter the latest. The facultative type has a physical deletion associated with the VRN-H2 gene causing loss of repression of the winter allele VRN-H127. In winter cultivars, vernalization requirement is given by the presence of the recessive VRN-H1 allele along with the dominant VRN-H2 allele (Rizza et al., 2016).

The facultative barley group reached heading date from 86 to 214 days after sowing, depending on the sowing date (Figure 2A). Within this group, if the genetic type *abbb* is used as a benchmark, the different number of days to heading is about +7/-2 days with differences increasing for later sowing dates, especially for the genetic type *abaa* (Supplementary Figure 3). The genetic type *abaa* has the vernalization (VRN-H2) allele recessive and photoperiod (PPD-H2) and earliness per se (EPS2) dominant. All the other genetic types with either photoperiod or earliness recessive showed values close to the type (*abbb*) with both recessive PPD-H2 and EPS2 (Figure 2A and Supplementary Figure 3).

Overall, spring barley genotypes did not show marked differences in the mean response to heading dates, but later planting in Algeria, Jordan, and Syria caused a delay in heading date and an exposure to terminal (heat+drought) stresses. The optimal window for heading date varies among locations, with some, like northern Italy (Fiorenzuola, Figure 2B), showing a wider window with respect to Syria (Figure 2B). In the latter location, the optimal window is smaller due to the earlier impacts of higher maximum air temperature. Within spring barley, the main commonality between the allelic combinations is the VRN-H1 being dominant. The difference in heading date between each spring genetic type and the *bbbb* (taken as benchmark) is showed in Supplementary Figure 3. An interesting result is that the genetic type *baaa* that has the VRN-H2 dominant allele as well as photoperiod and earliness had up to -11 days of heading dates respect to the *bbbb* (Supplementary Figure 3). As in the case of the facultative barley, sowing dates from October 30 to December 15 showed lower differences with respect to the later sowing (Figure 2B and Supplementary Figure 3).

The winter genotypes also showed the same type of response to planting date as the spring ones (Figure 2C). In one location, Foggia (Italy), the different allelic combinations were at the edge of the optimal heading window for late planting. Only in Fiorenzuola (Italy), Spain, and Turkey did all the sowing dates have the heading time within the optimal window (Figure 2C). For the winter types the *aaaa* allelic combination was used as a benchmark, but the differences with respect to this combination were maximum 2 days and there was not a marked difference among allelic combinations (Supplementary Figure 3). Another comparison was made, at each location and for each allelic combination, against the two parent cultivars (NURE and TREMOIS). In this case, there was an even higher variability in heading dates with the facultative barley types having up to 15 days early heading dates with respect to NURE and TREMOIS (Supplementary Figure 4).

### Climate Impacts on Heading Date

**FIGURE 3:**
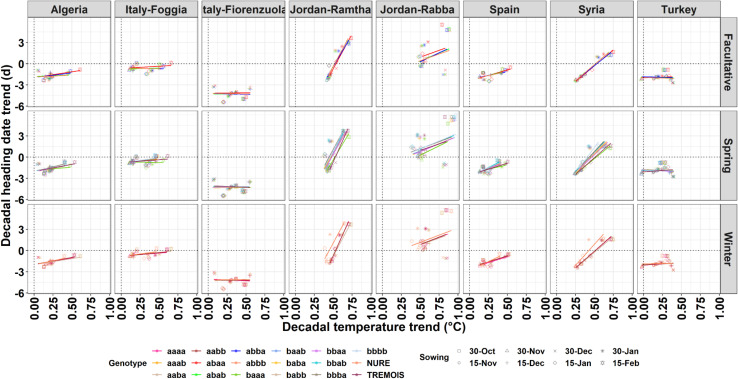
Relationship between the decadal temperature trend and decadal heading date for the different genotypes (different line colors) and different sowing dates (PlDate, different shapes) for the facultative, spring, and winter barley types.

To quantify the impacts of the long-term air temperature trends, the decadal mean air temperature changes are plotted against the decadal heading date trends for each location, genotype, and planting date (Figure 3). The mean air temperature increases between 0.1°C and 0.75°C per decade across the different locations, with greater increase for later planting dates. The decadal heading date trends range between −3 and + 3 days per decade (Figure 3). Some locations, like Jordan-Ramtha, showed a steep increase in days per decade as the decadal temperature increased, and this was true across planting dates and genotypes, while in all others there is still a clear separation of response by genotypes (Figure 3).

When the same analysis is done by considering the impacts of different planting dates, the changes in number of days to heading per degree increase in temperature varies across locations and genotypes (Supplementary Figure 5). The number of days to heading per degree increase in temperature varies between −10 and +6, with cooler locations such as northern Italy (Fiorenzuola) or the location in Turkey showing lower variability across dates and genotypes (Supplementary Figure 5).

The climate of the locations in terms of maximum and minimum temperature showed that north Italy (Fiorenzuola) and Turkey had the lowest minimum air temperature (Figure 1). The growing season daylength differs across locations and explains the different response of heading date to temperature at different locations (Supplementary Figure 6). Planting dates also impact the growing season day length, going from a 12 h/day for the early planting to 15 h/day for the late planting dates (Supplementary Figure 6). There is also a difference among the different allelic combinations, with the facultative and spring types showing slightly higher average daylength (expressed in hours/day) from sowing to harvest (Supplementary Figure 6).

### Effects of Planting Date and Fertilizer Management on Yield

**FIGURE 4:**
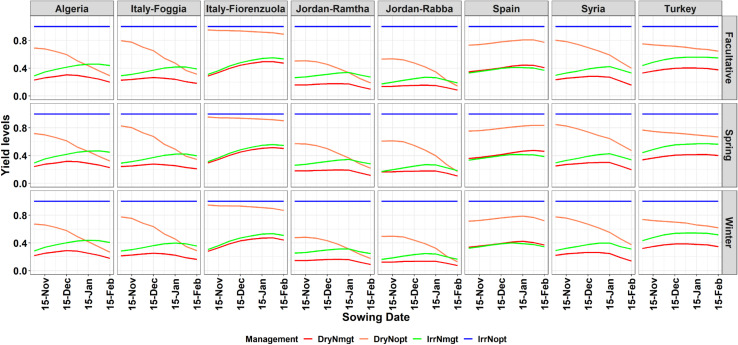
Yield gap between the different barley types simulated with optimal managements conditions (no water or nitrogen stresses; **IrrNopt**, blue top straight line), no water stress and current nitrogen management (**IrrNmgt**, green line), dryland and optimal nitrogen management (**DryNopt**, orange line), and under current management conditions (**DryNmgt**, red line).

The yield gap between potential and actual yield is mostly due to nitrogen management across locations and genotypes (Figure 4). There is a difference between the three different types of barley on how the yield changes for the different combinations of water/nitrogen at different planting dates. The horizontal blue line, equal to one, is the condition in which a potential yield can be reached. The nitrogen management applied under rainfed conditions (**DryNmgt**, red line in Figure 4) produced lower yield respect to the other combinations, and within its own range, it produced better yields for late-Nov/mid-Dec planting dates (Figure 4). An optimal N management, where N could potentially be applied at key stages to avoid stress (**Dry Nopt**, orange line in Figure 4) caused an increase in yield, and in locations like northern Italy (Fiorenzuola) yield reaches levels close to the potential. Interestingly, such management optimizes yield for early planting dates, and decreases to levels close to the actual management for later planting (Figure 4). The additional deficit irrigation technique with the current nitrogen management (**IrrNmgt**; green line in Figure 4) has a similar pattern of the red line (dry and current nitrogen management) but higher yield levels, especially for later planting dates. The simulated yield does not decrease with later planting but reached a plateau (Figure 3). While the differences among different allelic combinations are shown in Supplementary Figures 7–9 and show little yield gains/loss for different combinations. The absolute yield levels simulated under dry conditions and for the 30 years showed some variability among the genotypes, with allelic combinations that reached later heading dates having lower simulated yield than the others (Supplementary Figure 10). There is also a variability in terms of sowing dates, with high maximum absolute yield obtained for the December planting while later sowing dates showed lower absolute yield. There was a variability among sites with Turkey showing little variability in terms of yield response to planting date while Fiorenzuola showed an increase in yield with later planting dates up to 15 Jan (Supplementary Figure 10).

## Discussion

Genetic characterization of pivotal genes that can discriminate between growth types, earliness, photoperiod response, plant height, major resistances, and other major traits in economically important cereals is increasing in the last decades in an even larger sample of cultivated germplasm. This information does not only constitute a benchmark for the cereal breeding strategies, but should also be more diffusely implemented in agronomic models, to modulate management as to improve precision of yield predictions. In the Mediterranean basin there has been an increase in mean temperature of 0.50°C per decade over the 1980–2010, with projections showing a further temperature increase between 0.90°C and 2.16°C by mid of the Century (Cammarano et al., 2019a).

An important practical aspect is the identification of the optimal sowing date and genotype that would escape late frost and heat stresses in the period around the heading date, a growth stage that influences the grain number per spike (Zheng et al., 2013). In a previous study on the same genotypes there were no indication about this risk, although the authors acknowledge it (Francia et al., 2011). The gene-based calibration of crop models has been achieved in several ways, depending on the details available on the experimental data. Zheng et al. (2013) achieved to quantify the effects of VRN1 and ppd-D1 to predict spring wheat across multi-environments, and they did so by obtaining data in order to control vernalization and photoperiod and achieve a detailed gene-based calibration. On the other hand, White et al. (2008) obtained a gene-based calibration from only anthesis date on wheat. Results uses the latter approach and brings it a step forward by considering the effects of VRN-H1, VRN-H2, PPD-H2, and EPS2 related to agronomic combination of sowing date, nitrogen management, and the risk of frost/heat on heading dates.

The impacts of low temperature and day length on the expression of Vrn and Ppd loci to adapt the growth cycle to different cultivation areas are well known and reported in several studies (von Zitzewitz et al., 2005; Francia et al., 2011). The impacts of temperature changes on barley heading time indicated that for some locations such as Fiorenzuola (north Italy) the changes in decadal temperatures were less important, because the main effect was ascribed to the growing day length. As the growing season daylength increased, their response to increased temperature decreased confirming the results showed in north Europe (Cammarano et al., 2019b). The results of our study regarding the environmental impacts on barley phenology agrees with the molecular study findings reported elsewhere (Francia et al., 2011) that did not consider the earliness per se. In barley, most of the growth habit is regulated by the combinations of VRN-H1 and VRN-H2 and within the same allelic combination of these two Vrn drivers, the photoperiod can modify the length of the cycle (von Zitzewitz et al., 2005). These experimental findings were also confirmed in our study, showing how a gene-based model calibration is useful in characterizing the different barley genotypes. On the one hand, this suggests that by including few genetic information into a crop growth model it is possible to simulate a GxExM simply using phenological genes (Vrn, Ppd, and Eam). It is useful to point out that other photoperiod and vernalization genes (e.g., Ppd1 and Vrn3) were not segregating in the barley population studied in our work. Another point to highlight is that the NURE x TREMOIS barley genotypes used in this study segregated for frost resistance. However, results of this study showed that the effect of frost is not impacting any location and genotype.

The accuracy of representing heading date using allelic information agrees with the results showed by White et al. (2008) with respect to the manual calibration/evaluation. In a previous study the RMSE was between 6 and 10 days for the manual and gene-based calibration (White et al., 2008) and in our study the RMSE decreased from 8 to 7 days for the gene-based approach. However, this study also compared simulated maturity and yield data showing that a slight improvement was evident.

The novelty of the approach presented here with respect to the study of White et al. (2008) is that it took into account the impact of the earliness per se EPS2 gene through the modification of the period between the end of juvenile to terminal spikelet stage. It was argued that some of the inaccuracy of the gene-based calibration on wheat was the inability for accounting the interactions between VRN-H1 and Ppd-D1 loci (Zheng et al., 2013). In this study, the interactions between VRN-H1 and VRN-H2 and not with the PPD-H2 is supported by the current experimental evidence (von Zitzewitz et al., 2005). Results of the present study showed that for the same vernalization combinations, photoperiod and earliness per se modify the length of the cycle, and this is more evident when the VRN-H2 is recessive (Figure 2). An experimental study found similar results on the same locations and with the same genotypes, and agrees with the same conclusions on the importance of few phenology determinants on a complex trait such as barley grain yield (Francia et al., 2011). In hot environments such as Algeria, Syria, and Jordan early sowing dates (October 30 and December 15) would be chosen to minimize the risk of exposing barley to heat stress. For the Mediterranean basin, barley breeding activities should focus on allelic combinations that have recessive VRN-H2 and dominant earliness per se, since the risk of cold stress is much lower than the heat stress. This information might be useful to develop all elite genotypes suitable to grow and yield in the Mediterranean Basin (Monteagudo et al., 2019). It was also found that the facultative genetic types show similar or higher frost resistance than winter genotypes under suboptimal hardening conditions (Rizza et al., 2016).

The yield analysis of our study demonstrated how optimal nitrogen management could help reduce the yield gap, and how sowing dates in the mid of the simulated window optimize both heading date (within the optimal window) and grain yield. Mediterranean environments show a high inter-annual variability of annual rainfall, with severe summer droughts and an unreliable amount of rainfall for the growing season which can cause a large variability in grain yield (Cammarano et al., 2019a). The actual grain yield and the simulated yield potential (**IrrNopt**) depend on the combination of the genotype, the environmental conditions, and the agronomic management (GxExM).

Nitrogen fertilization is the most critical agronomic input affecting barley production and farm profitability. However, the closing of the yield gap, for each genotype, in such an environment is challenging because of the dynamic interactions between soil water content, nitrogen uptake, and air temperature. In fact, considering only one GxE, some agronomic interactions might be missed between soil and the plant that would impact on growth and yield. Crop growth is related to the amount of soil water, rainfall, solar radiation, and nutrient availability. These factors interact among each other, for example roots uptake water and nutrients and the canopy captures solar radiation and atmospheric CO_2_ concentration and transforms these into biomass (Jamieson and Ewert, 1999; Sadras and Angus, 2006). By linking the gene effects for phenology with a process-based model (that considers the soil-plant-atmosphere-agronomy relationships) the whole system is not considered as a vacuum. But some of these interactions become evident as shown also in previous studies (Cammarano et al., 2019a). In fact, the impacts of temperature are not considered on their own, but they interact with location-specific soil types and genotype-specific crops. The soil of this study was used in a previous work and was characterized for each location (Cammarano et al., 2019a). This is an important thing to point out for explaining the results of Figure 4. To close the gap, optimal nitrogen amounts should be tactically and strategically applied to optimize the nitrogen uptake without losing all the water that could be used later in the growing season (Sadras and Angus, 2006). This can be achieved by adopting several agronomic practices such as shifting of sowing dates for escaping heat stresses and terminal droughts. In addition, the closing of the gap can be obtained by adoptions of site-specific nitrogen management (Cammarano et al., 2020). It has been shown that there is a negative correlation between grain yield and days to heading under drought conditions (Ceccarelli et al., 1998). Finding the best combinations of alleles and agronomic management allow the crops to escape heat stresses to complete their cycle while water is still available.

In conclusion, the adaptation of barley genotypes and agronomic management to climate variability, and its impact on production are important. It would be useful to do the same study using projected climate in order to inform breeding strategies. Results of this study show some promising ways of simply integrating phenological genes effects into crop growth models and giving useful agronomic (sowing date and N fertilization) and genetic (traits to develop elite genotype) information for the sustainable production of barley in the Mediterranean basin. Since an increasing number of cultivated germplasms in wheat and barley is characterized with an impressively increasing amount of sequence diversity data, further studies might investigate which other genes, and how many characterized genes, would be worth including into agronomic models until reaching a trade-off of information input vs. output.

## Data Availability Statement

The original contributions presented in the study are included in the article/Supplementary Material, further inquiries can be directed to the corresponding author.

## Author Contributions

DC ran the crop model, analyzed the data, and wrote the manuscript. DR calibrated and evaluated the crop model and reviewed the manuscript. EF wrote, reviewed, and edited the manuscript. TA, AA-Y, AB, SG, IR, and AS generated the data. NP conceived and promoted the present study, and reviewed and edited the manuscript. All authors contributed to the article and approved the submitted version.

## Conflict of Interest

The authors declare that the research was conducted in the absence of any commercial or financial relationships that could be construed as a potential conflict of interest.
