# Supplementary material for: Genetic and Management Effects on Barley Yield and Phenology in the Mediterranean Basin
Source: Front Plant Sci. 2021 Apr 15;12:655406. doi: 10.3389/fpls.2021.655406 (PMC8084452; doi:10.3389/fpls.2021.655406)
Supplement: Supplementary file 1 [file Data_Sheet_1.docx]

Supplementary Material

Genetic and management effects on barley yield and phenology in the Mediterranean basin

Davide Cammarano^1*^, Domenico Ronga^2,3^, Enrico Francia^2^, Taner Akar^4^, Adnan Al-Yassin^5^, Abdelkader Benbelkacem^6^, Stefania Grando^7^, Ignacio Romagosa^8^, Antonio Michele Stanca^2^, and Nicola Pecchioni^9^

^1^Department of Agronomy, Purdue University, 915 W. State Street, West Lafayette, IN 47907, USA

^2^Department of Life Science, Centre BIOGEST-SITEIA, University of Modena and Reggio Emilia, Via Amendola, n. 2, 42122 Reggio Emilia (RE), Italy

^3^Pharmacy Department, University of Salerno, Via Giovanni Paolo II n.132, 84084 Fisciano (SA), Italy

^4^Akdeniz University, Faculty of Agriculture, Department of Agronomy, Antalya, Turkey

^5^National Agricultural Research Center (NCARE), Amman, Jordan

^6^National Agronomic Research Institute of Algeria (INRAA), Constantine, Algeria

^7^International consultant, Italy

^8^Agrotecnio Centre, Universitat de Lleida, Lleida, Spain

^9^Research Centre for Cereal and Industrial Crops, CREA - Council for Agricultural Research and Economics, S.S. 673 Km 25200, 71122 Foggia, Italy

***Correspondence:**Davide Cammarano
dcammar@purdue.edu

Keywords: Crop modeling, Barley, Genotypes, management, Mediterranean.

**Supplemental Table 1.** Allelic information and DSSAT model parameters for each of the genetic type.

|  |  | **Locus** | | | | **Model parameters** | |  |
| --- | --- | --- | --- | --- | --- | --- | --- | --- |
| **Habit** | **alleles** | **VrnH1** | **VrnH2** | **PpdH2** | **EPS2** | **P1V** | **P1D** | **P1** |
| - | - | - | - | - | - | **d** | **%** | ***PVTU*** |
| Facultative | *abbb* | 1 | 0 | 0 | 0 | 34 | 77 | 380 |
| Facultative | *abab* | 1 | 0 | 1 | 0 | 34 | 79 | 380 |
| Facultative | *abba* | 1 | 0 | 0 | 1 | 34 | 77 | 305 |
| Facultative | *abaa* | 1 | 0 | 1 | 1 | 34 | 79 | 305 |
| Spring | *bbbb* | 0 | 0 | 0 | 0 | 27 | 65 | 350 |
| Spring | *babb* | 0 | 1 | 0 | 0 | 23 | 77 | 380 |
| Spring | *baab* | 0 | 1 | 1 | 0 | 25 | 79 | 380 |
| Spring | *bbab* | 0 | 0 | 1 | 0 | 23 | 79 | 380 |
| Spring | *bbaa* | 0 | 0 | 1 | 1 | 23 | 79 | 305 |
| Spring | *baaa* | 0 | 1 | 1 | 1 | 25 | 79 | 305 |
| Spring | *bbba* | 0 | 0 | 0 | 1 | 23 | 77 | 305 |
| Spring | *baba* | 0 | 1 | 0 | 1 | 25 | 77 | 305 |
| Winter | *aabb* | 1 | 1 | 0 | 0 | 48 | 77 | 380 |
| Winter | *aaaa* | 1 | 1 | 1 | 1 | 48 | 79 | 305 |
| Winter | *aaab* | 1 | 1 | 1 | 0 | 48 | 79 | 380 |
| Winter | *aaba* | 1 | 1 | 0 | 1 | 48 | 77 | 305 |
| Winter | *NURE* | 1 | 1 | 1 | 1 | 48 | 79 | 305 |
| Spring | *TREMOIS* | 0 | 0 | 0 | 0 | 23 | 77 | 380 |

**Supplemental Table 2.** Site description in terms of locations (coordinates and altitude), mean seasonal air minimum and maximum temperature, the type of field experiment (if rainfed or irrigated), the available water content the water input and the mean recorded grain yield across all the barley genetic types. Modified from previous published work (Francia et al., 2011; Cammarano et al., 2019).

| **ID** | | **Site** | | **Latitude** | | **Longitude** | | **Altitude** | | **T*min^a^*** | | **T*max^b^*** | **Type** | **AWC**^c^ | **Water Input**^d^ | **Grain Yield**  **(t ha^-1^)** |
| --- | --- | --- | --- | --- | --- | --- | --- | --- | --- | --- | --- | --- | --- | --- | --- | --- |
| ALGA | | El Khroub | | 36°15’N | | 06°42’E | | 596 | | 8.9 | | 22.4 | Rainfed | 100 | 130 | 3.50 |
| ITAA | | Foggia | | 41°28’N | | 15°33’E | | 57 | | 5.9 | | 16.7 | Irrigated | 130 | 327 | 3.78 |
| ITAF | | Foggia | | 41°28’N | | 15°33’E | | 57 | | 7.0 | | 17.8 | Rainfed | 130 | 268 | 3.85 |
| ITAG | | Foggia | | 41°28’N | | 15°33’E | | 57 | | 5.9 | | 16.3 | Rainfed | 130 | 258 | 3.20 |
| ITAH | | Foggia | | 41°28’N | | 15°33’E | | 57 | | 7.0 | | 17.8 | Irrigated | 130 | 362 | 4.88 |
| ITAL | | Fiorenzuola | | 44°55’N | | 09°54’E | | 80 | | 2.8 | | 15.0 | Rainfed | 144 | 292 | 4.58 |
| JORA | | Ramtha | | 32°32’N | | 36°02’E | | 561 | | 7.2 | | 20.2 | Rainfed | 120 | 151 | 1.33 |
| JORB | | Ramtha | | 32°32’N | | 36°02’E | | 561 | | 7.4 | | 22.1 | Rainfed | 120 | 140 | 0.50 |
| JORC | | Rabba | | 31°16’N | | 35°44’E | | 616 | | 6.3 | | 18.6 | Rainfed | 120 | 194 | 0.07 |
| JORD | | Rabba | | 31°16’N | | 35°44’E | | 616 | | 7.0 | | 18.7 | Rainfed | 120 | 217 | 0.80 |
| SPAA | | Foradada | | 41°39’N | | 01°23’W | | 318 | | 1.7 | | 15.8 | Rainfed | 120 | 167 | 0.48 |
| SYRA | | Breda | | 35°56’N | | 37°10’E | | 300 | | 7.5 | | 20.0 | Rainfed | 153 | 204 | 1.35 |
| SYRB | | Breda | | 35°56’N | | 37°10’E | | 300 | | 7.0 | | 20.4 | Rainfed | 153 | 143 | 2.42 |
| TURA | | Haymana | | 39°26’N | | 32°30’E | | 1214 | | 5.8 | | 16.2 | Rainfed | 150 | 232 | 3.30 |
| TURB | | Haymana | | 39°26’N | | 32°30’E | | 1214 | | 5.8 | | 16.2 | Irrigated | 150 | 282 | 4.44 |
| TURC | | Haymana | | 39°26’N | | 32°30’E | | 1214 | | 12.6 | | 25.6 | Rainfed | 150 | 174 | 3.89 |
| *^a^Maximum air temperature (°C);*  *^b^Minimum air temperature (°C);*  *^c^Available water holding capacity (mm m^-1^) of the soil;*  *^d^Total rainfall plus irrigation (mm) from sowing to harvest.* | | | | | | | | | |  | |  |  |  |  |  |
|  |  | |  | |  | |  | |  | |  |  |  |  |  |  |
|  |  | |  | |  | |  | |  | |  |  |  |  |  |  |

**Supplemental Table 3.** Observed heading date at each site and for each genetic type.

| **Location** | **Allels** | **Observed heading (d)** |  | **Location** | **Allels** | **Observed heading (d)** |  | **Location** | **Allels** | **Observed heading (d)** |
| --- | --- | --- | --- | --- | --- | --- | --- | --- | --- | --- |
| Algeria - El Khroub | aaaa | 96 |  | Italy - Fiorenzuola | abab | 139 |  | Italy - Fiorenzuola | baba | 134 |
| Algeria - El Khroub | aaab | 96 |  | Italy - Fiorenzuola | abba | 134 |  | Italy - Fiorenzuola | babb | 136 |
| Algeria - El Khroub | aaba | 96 |  | Italy - Fiorenzuola | abbb | 139 |  | Italy - Fiorenzuola | bbaa | 134 |
| Algeria - El Khroub | aabb | 96 |  | Italy - Fiorenzuola | baaa | 135 |  | Italy - Fiorenzuola | bbab | 137 |
| Algeria - El Khroub | abaa | 96 |  | Italy - Fiorenzuola | baab | 139 |  | Italy - Fiorenzuola | bbba | 133 |
| Algeria - El Khroub | abab | 98 |  | Italy - Fiorenzuola | baba | 135 |  | Italy - Fiorenzuola | bbbb | 137 |
| Algeria - El Khroub | abba | 96 |  | Italy - Fiorenzuola | babb | 138 |  | Italy - Fiorenzuola | NURE | 135 |
| Algeria - El Khroub | abbb | 96 |  | Italy - Fiorenzuola | bbaa | 135 |  | Italy - Fiorenzuola | TREMOIS | 137 |
| Algeria - El Khroub | baaa | 95 |  | Italy - Fiorenzuola | bbab | 140 |  | Italy - Fiorenzuola | aaaa | 175 |
| Algeria - El Khroub | baab | 96 |  | Italy - Fiorenzuola | bbba | 134 |  | Italy - Fiorenzuola | aaab | 180 |
| Algeria - El Khroub | baba | 95 |  | Italy - Fiorenzuola | bbbb | 139 |  | Italy - Fiorenzuola | aaba | 173 |
| Algeria - El Khroub | babb | 95 |  | Italy - Fiorenzuola | NURE | 133 |  | Italy - Fiorenzuola | aabb | 181 |
| Algeria - El Khroub | bbaa | 96 |  | Italy - Fiorenzuola | TREMOIS | 139 |  | Italy - Fiorenzuola | abaa | 173 |
| Algeria - El Khroub | bbab | 96 |  | Italy - Fiorenzuola | aaaa | 115 |  | Italy - Fiorenzuola | abab | 181 |
| Algeria - El Khroub | bbba | 95 |  | Italy - Fiorenzuola | aaab | 119 |  | Italy - Fiorenzuola | abba | 172 |
| Algeria - El Khroub | bbbb | 96 |  | Italy - Fiorenzuola | aaba | 112 |  | Italy - Fiorenzuola | abbb | 180 |
| Algeria - El Khroub | NURE | 97 |  | Italy - Fiorenzuola | aabb | 116 |  | Italy - Fiorenzuola | baaa | 177 |
| Algeria - El Khroub | TREMOIS | 95 |  | Italy - Fiorenzuola | abaa | 113 |  | Italy - Fiorenzuola | baab | 182 |
| Italy - Fiorenzuola | aaaa | 120 |  | Italy - Fiorenzuola | abab | 117 |  | Italy - Fiorenzuola | baba | 175 |
| Italy - Fiorenzuola | aaab | 123 |  | Italy - Fiorenzuola | abba | 111 |  | Italy - Fiorenzuola | babb | 179 |
| Italy - Fiorenzuola | aaba | 118 |  | Italy - Fiorenzuola | abbb | 116 |  | Italy - Fiorenzuola | bbaa | 175 |
| Italy - Fiorenzuola | aabb | 121 |  | Italy - Fiorenzuola | baaa | 112 |  | Italy - Fiorenzuola | bbab | 182 |
| Italy - Fiorenzuola | abaa | 117 |  | Italy - Fiorenzuola | baab | 115 |  | Italy - Fiorenzuola | bbba | 174 |
| Italy - Fiorenzuola | abab | 123 |  | Italy - Fiorenzuola | baba | 111 |  | Italy - Fiorenzuola | bbbb | 182 |
| Italy - Fiorenzuola | abba | 117 |  | Italy - Fiorenzuola | babb | 113 |  | Italy - Fiorenzuola | NURE | 173 |
| Italy - Fiorenzuola | abbb | 122 |  | Italy - Fiorenzuola | bbaa | 112 |  | Italy - Fiorenzuola | TREMOIS | 184 |
| Italy - Fiorenzuola | baaa | 115 |  | Italy - Fiorenzuola | bbab | 116 |  | Jordan - Ramtha | aaaa | 112 |
| Italy - Fiorenzuola | baab | 121 |  | Italy - Fiorenzuola | bbba | 110 |  | Jordan - Ramtha | aaab | 114 |
| Italy - Fiorenzuola | baba | 117 |  | Italy - Fiorenzuola | bbbb | 116 |  | Jordan - Ramtha | aaba | 108 |
| Italy - Fiorenzuola | babb | 118 |  | Italy - Fiorenzuola | NURE | 113 |  | Jordan - Ramtha | aabb | 112 |
| Italy - Fiorenzuola | bbaa | 118 |  | Italy - Fiorenzuola | TREMOIS | 114 |  | Jordan - Ramtha | abaa | 106 |
| Italy - Fiorenzuola | bbab | 122 |  | Italy - Fiorenzuola | aaaa | 135 |  | Jordan - Ramtha | abab | 115 |
| Italy - Fiorenzuola | bbba | 116 |  | Italy - Fiorenzuola | aaab | 138 |  | Jordan - Ramtha | abba | 107 |
| Italy - Fiorenzuola | bbbb | 121 |  | Italy - Fiorenzuola | aaba | 134 |  | Jordan - Ramtha | abbb | 113 |
| Italy - Fiorenzuola | NURE | 118 |  | Italy - Fiorenzuola | aabb | 136 |  | Jordan - Ramtha | baaa | 107 |
| Italy - Fiorenzuola | TREMOIS | 121 |  | Italy - Fiorenzuola | abaa | 135 |  | Jordan - Ramtha | baab | 111 |
| Italy - Fiorenzuola | aaaa | 137 |  | Italy - Fiorenzuola | abab | 137 |  | Jordan - Ramtha | baba | 104 |
| Italy - Fiorenzuola | aaab | 140 |  | Italy - Fiorenzuola | abba | 133 |  | Jordan - Ramtha | babb | 104 |
| Italy - Fiorenzuola | aaba | 135 |  | Italy - Fiorenzuola | abbb | 137 |  | Jordan - Ramtha | bbaa | 107 |
| Italy - Fiorenzuola | aabb | 139 |  | Italy - Fiorenzuola | baaa | 134 |  | Jordan - Ramtha | bbab | 110 |
| Italy - Fiorenzuola | abaa | 135 |  | Italy - Fiorenzuola | baab | 137 |  | Jordan - Ramtha | bbba | 104 |

**Supplemental Table 4.** Observed maturity date at each site and for each genetic type.

| **Location** | **Allels** | **Observed maturity (d)** |  | **Location** | **Allels** | **Observed maturity (d)** |  | **Location** | **Allels** | **Observed maturity (d)** |
| --- | --- | --- | --- | --- | --- | --- | --- | --- | --- | --- |
| Jordan - Ramtha | bbbb | 113 |  | Syria - Breda | aaba | 122 |  | Turkey - Haymana | abbb | 206 |
| Jordan - Ramtha | NURE | 105 |  | Syria - Breda | aabb | 127 |  | Turkey - Haymana | baaa | 201 |
| Jordan - Ramtha | TREMOIS | 115 |  | Syria - Breda | abaa | 123 |  | Turkey - Haymana | baab | 210 |
| Jordan - Rabba | aaaa | 96 |  | Syria - Breda | abab | 131 |  | Turkey - Haymana | baba | 204 |
| Jordan - Rabba | aaab | 100 |  | Syria - Breda | abba | 121 |  | Turkey - Haymana | babb | 202 |
| Jordan - Rabba | aaba | 96 |  | Syria - Breda | abbb | 128 |  | Turkey - Haymana | bbaa | 205 |
| Jordan - Rabba | aabb | 98 |  | Syria - Breda | baaa | 124 |  | Turkey - Haymana | bbab | 207 |
| Jordan - Rabba | abaa | 96 |  | Syria - Breda | baab | 128 |  | Turkey - Haymana | bbba | 202 |
| Jordan - Rabba | abab | 99 |  | Syria - Breda | baba | 122 |  | Turkey - Haymana | bbbb | 206 |
| Jordan - Rabba | abba | 95 |  | Syria - Breda | babb | 124 |  | Turkey - Haymana | NURE | 191 |
| Jordan - Rabba | abbb | 99 |  | Syria - Breda | bbaa | 125 |  | Turkey - Haymana | TREMOIS | 209 |
| Jordan - Rabba | baaa | 93 |  | Syria - Breda | bbab | 130 |  | Turkey - Haymana | aaaa | 198 |
| Jordan - Rabba | baab | 97 |  | Syria - Breda | bbba | 121 |  | Turkey - Haymana | aaab | 205 |
| Jordan - Rabba | baba | 95 |  | Syria - Breda | bbbb | 127 |  | Turkey - Haymana | aabb | 198 |
| Jordan - Rabba | babb | 97 |  | Syria - Breda | NURE | 124 |  | Turkey - Haymana | abaa | 197 |
| Jordan - Rabba | bbaa | 96 |  | Syria - Breda | TREMOIS | 125 |  | Turkey - Haymana | abab | 210 |
| Jordan - Rabba | bbab | 98 |  | Syria - Breda | aaaa | 122 |  | Turkey - Haymana | abba | 197 |
| Jordan - Rabba | bbba | 94 |  | Syria - Breda | aaab | 126 |  | Turkey - Haymana | abbb | 206 |
| Jordan - Rabba | bbbb | 98 |  | Syria - Breda | aaba | 120 |  | Turkey - Haymana | baaa | 201 |
| Jordan - Rabba | NURE | 97 |  | Syria - Breda | aabb | 125 |  | Turkey - Haymana | baab | 210 |
| Jordan - Rabba | TREMOIS | 99 |  | Syria - Breda | abaa | 121 |  | Turkey - Haymana | baba | 204 |
| Spain - Foradada | aaaa | 176 |  | Syria - Breda | abab | 125 |  | Turkey - Haymana | babb | 202 |
| Spain - Foradada | aaab | 181 |  | Syria - Breda | abba | 119 |  | Turkey - Haymana | bbaa | 205 |
| Spain - Foradada | aaba | 175 |  | Syria - Breda | abbb | 125 |  | Turkey - Haymana | bbab | 207 |
| Spain - Foradada | aabb | 180 |  | Syria - Breda | baaa | 120 |  | Turkey - Haymana | bbba | 202 |
| Spain - Foradada | abaa | 177 |  | Syria - Breda | baab | 125 |  | Turkey - Haymana | bbbb | 206 |
| Spain - Foradada | abab | 180 |  | Syria - Breda | baba | 119 |  | Turkey - Haymana | NURE | 191 |
| Spain - Foradada | abba | 176 |  | Syria - Breda | babb | 122 |  | Turkey - Haymana | TREMOIS | 209 |
| Spain - Foradada | abbb | 179 |  | Syria - Breda | bbaa | 121 |  |  |  |  |
| Spain - Foradada | baaa | 177 |  | Syria - Breda | bbab | 126 |  |  |  |  |
| Spain - Foradada | baab | 181 |  | Syria - Breda | bbba | 119 |  |  |  |  |
| Spain - Foradada | baba | 176 |  | Syria - Breda | bbbb | 125 |  |  |  |  |
| Spain - Foradada | babb | 180 |  | Syria - Breda | NURE | 121 |  |  |  |  |
| Spain - Foradada | bbaa | 177 |  | Syria - Breda | TREMOIS | 123 |  |  |  |  |
| Spain - Foradada | bbab | 182 |  | Turkey - Haymana | aaaa | 198 |  |  |  |  |
| Spain - Foradada | bbba | 175 |  | Turkey - Haymana | aaab | 205 |  |  |  |  |
| Spain - Foradada | bbbb | 182 |  | Turkey - Haymana | aaba | 198 |  |  |  |  |
| Spain - Foradada | NURE | 175 |  | Turkey - Haymana | aabb | 206 |  |  |  |  |
| Spain - Foradada | TREMOIS | 180 |  | Turkey - Haymana | abaa | 197 |  |  |  |  |
| Syria - Breda | aaaa | 125 |  | Turkey - Haymana | abab | 210 |  |  |  |  |
| Syria - Breda | aaab | 129 |  | Turkey - Haymana | abba | 197 |  |  |  |  |

**
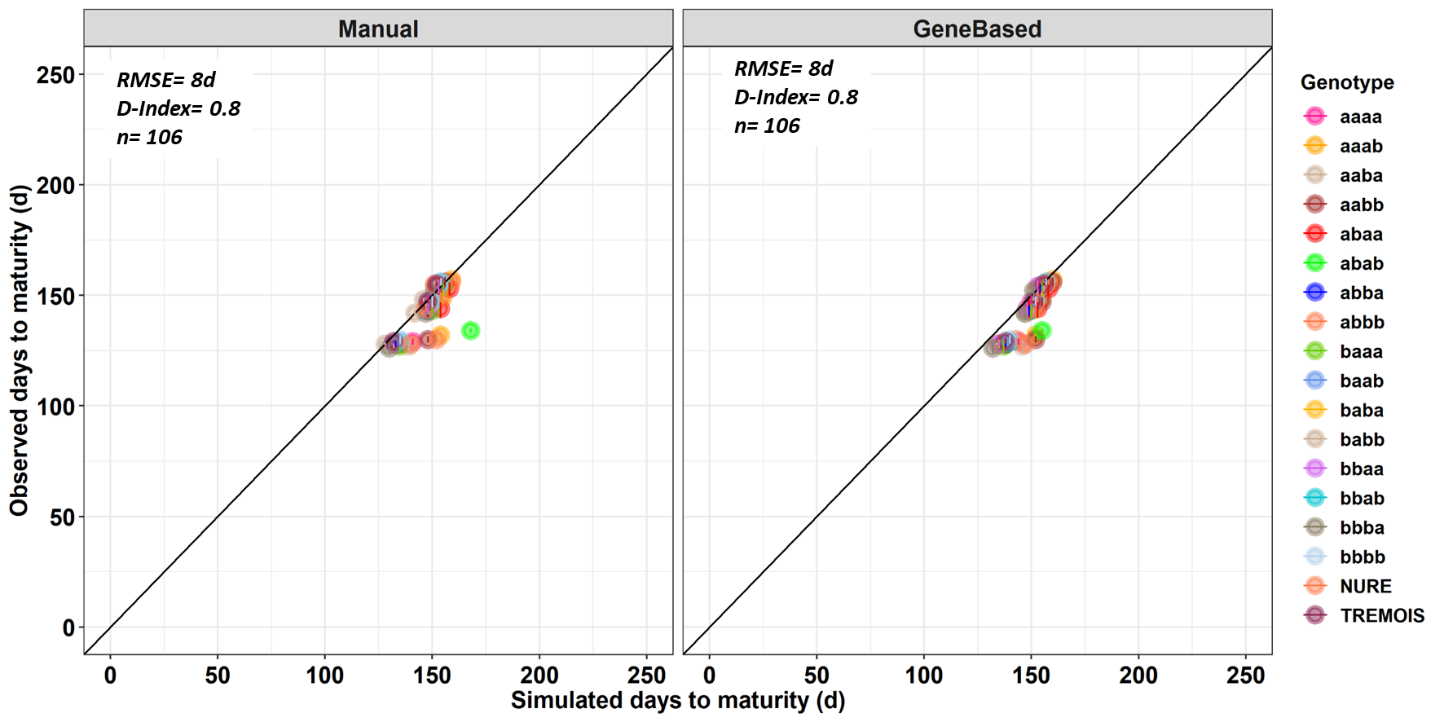
**

**Supplementary Figure 1.** Manual (left panel) and gene-based (right panel) fit against observed maturity dates for each of the eighteen genetic types. Error bars represent the standard deviation of number of days across the replicates and the lines used for each allelic combination.

**
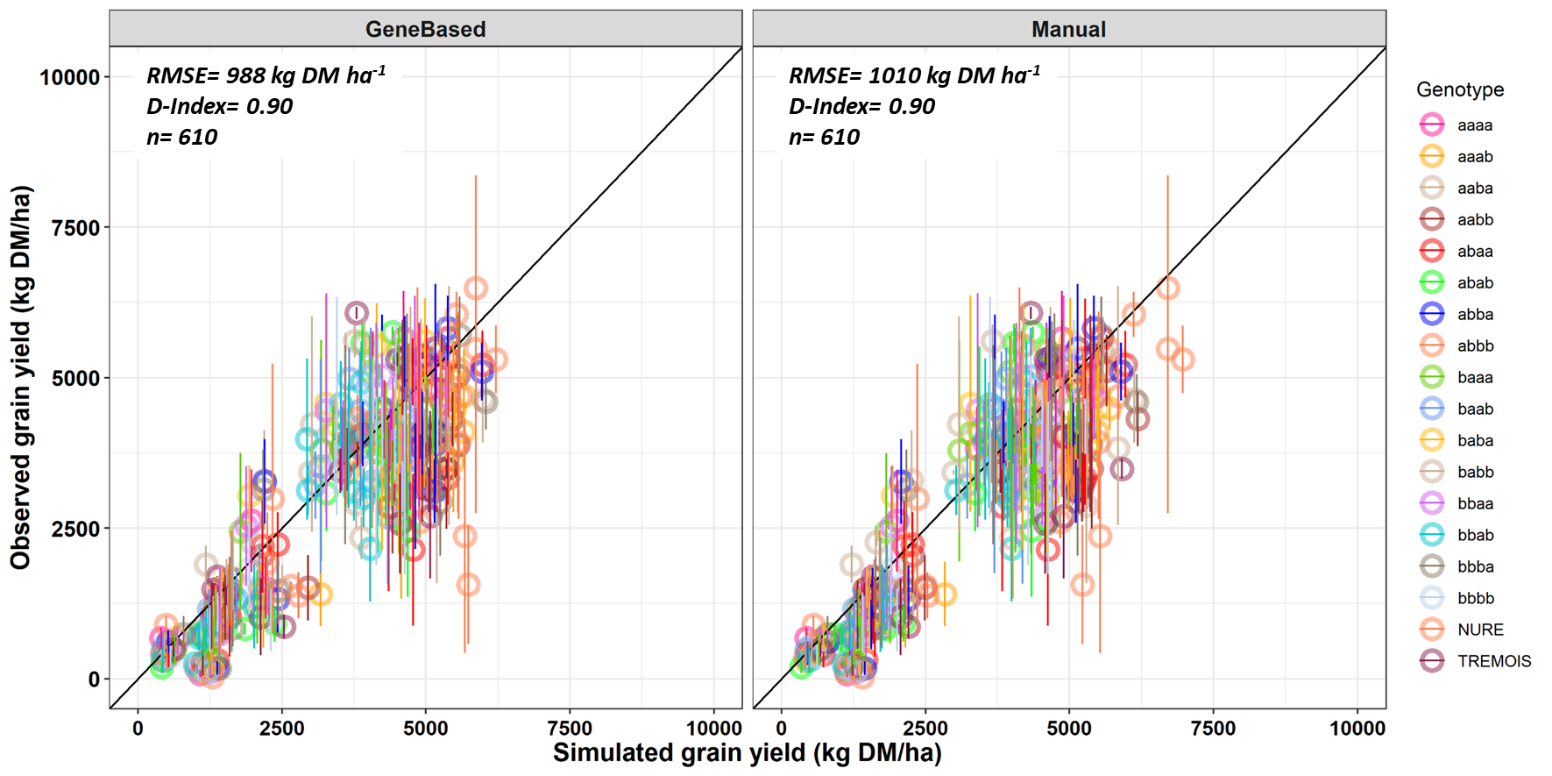
**

**Supplementary Figure 2.** Crop model calibration (left panel) and evaluation (right panel) fit against observed barley yield data for each of the eighteen genetic types. Error bars represent the standard deviation of number of yields across the replicates and the lines used for each allelic combination.

**
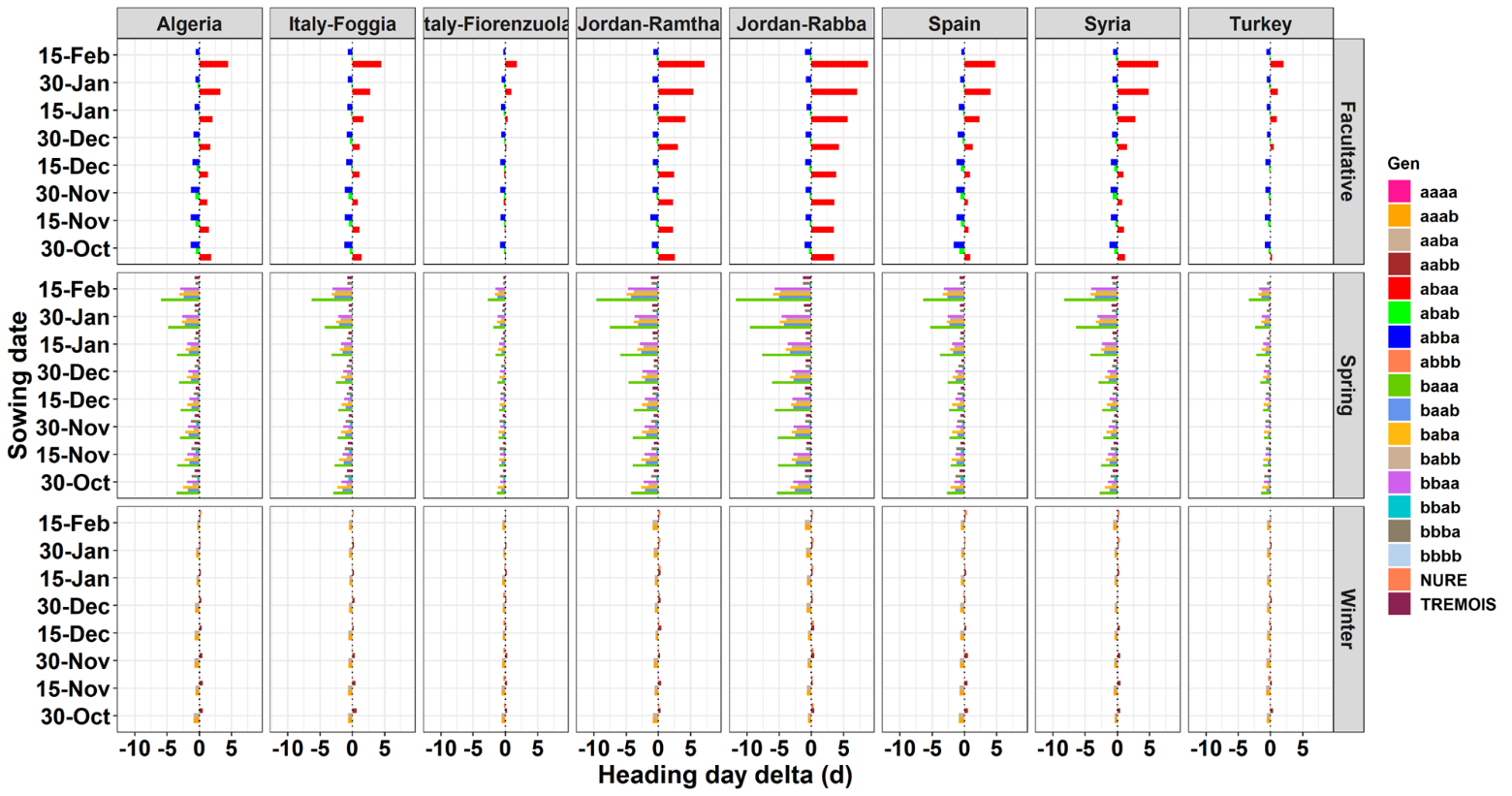
**

**Supplementary Figure 3.** Delta heading day between each genetic type and a genetic type taken as reference in each group. For the Facultative, Spring and Winter types the *abbb*, *bbbb*, and *aaaa* were taken as reference, respectively.


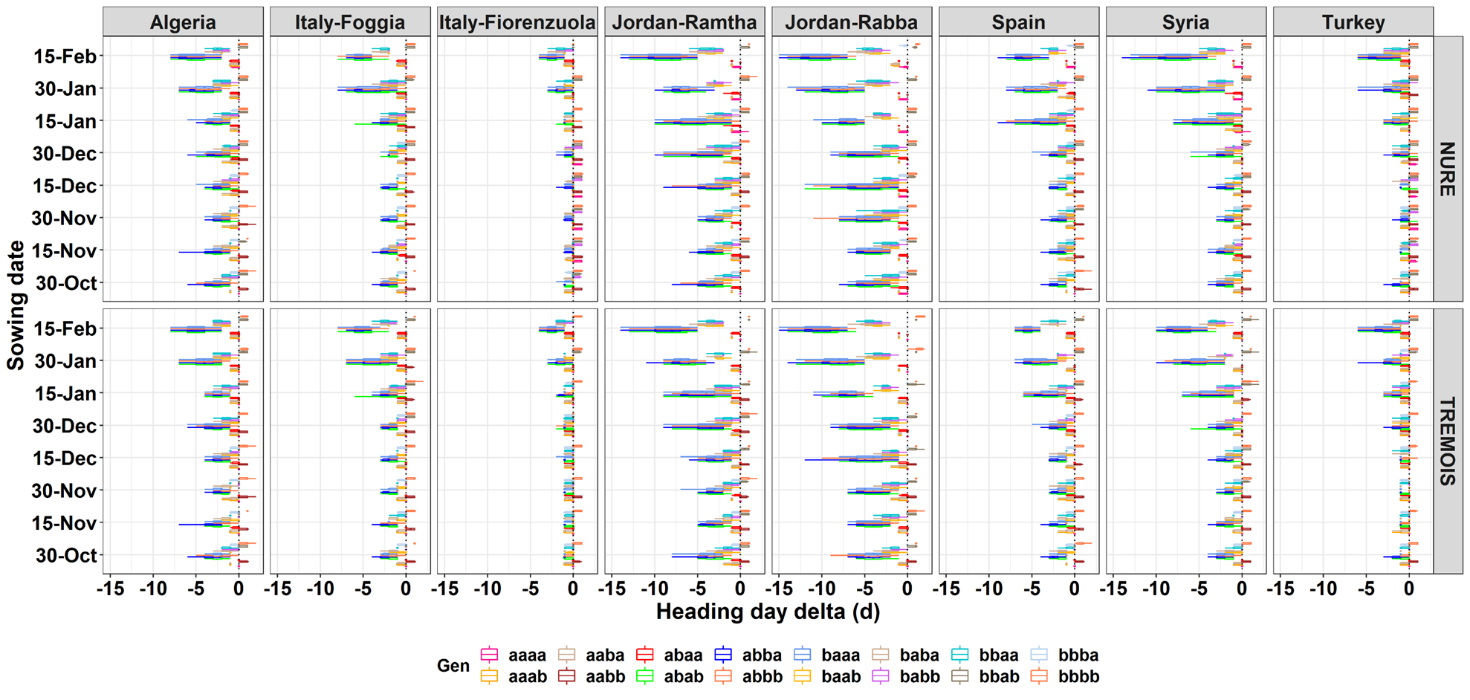


**Supplementary Figure 4.** The delta between each genetic type and either NURE (Top panels) and TREMOIS (bottom panels).


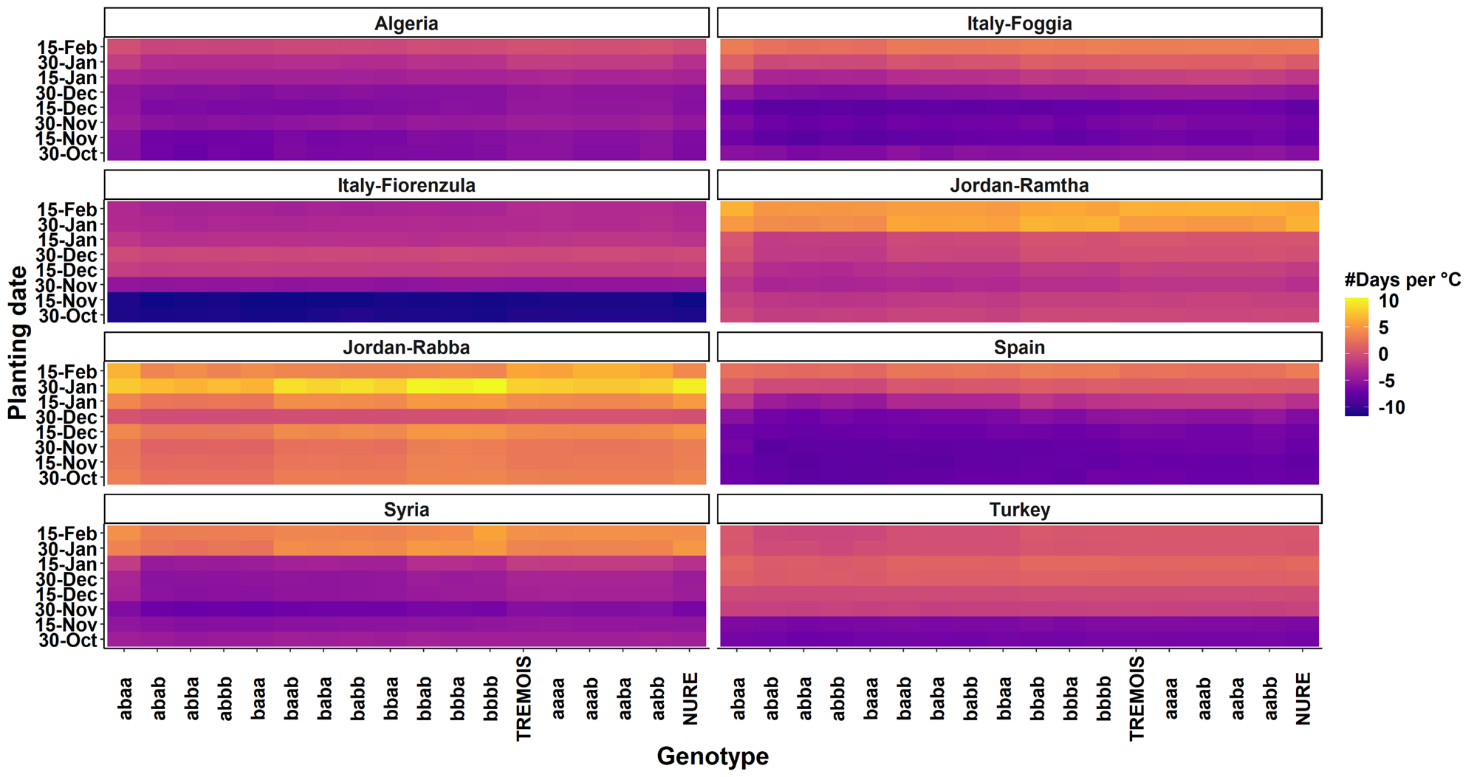


**Supplementary Figure 5.** Number of days per degree Celsius calculated from the simulated data using long-term weather data at each location, for each planting date and each genetic type.


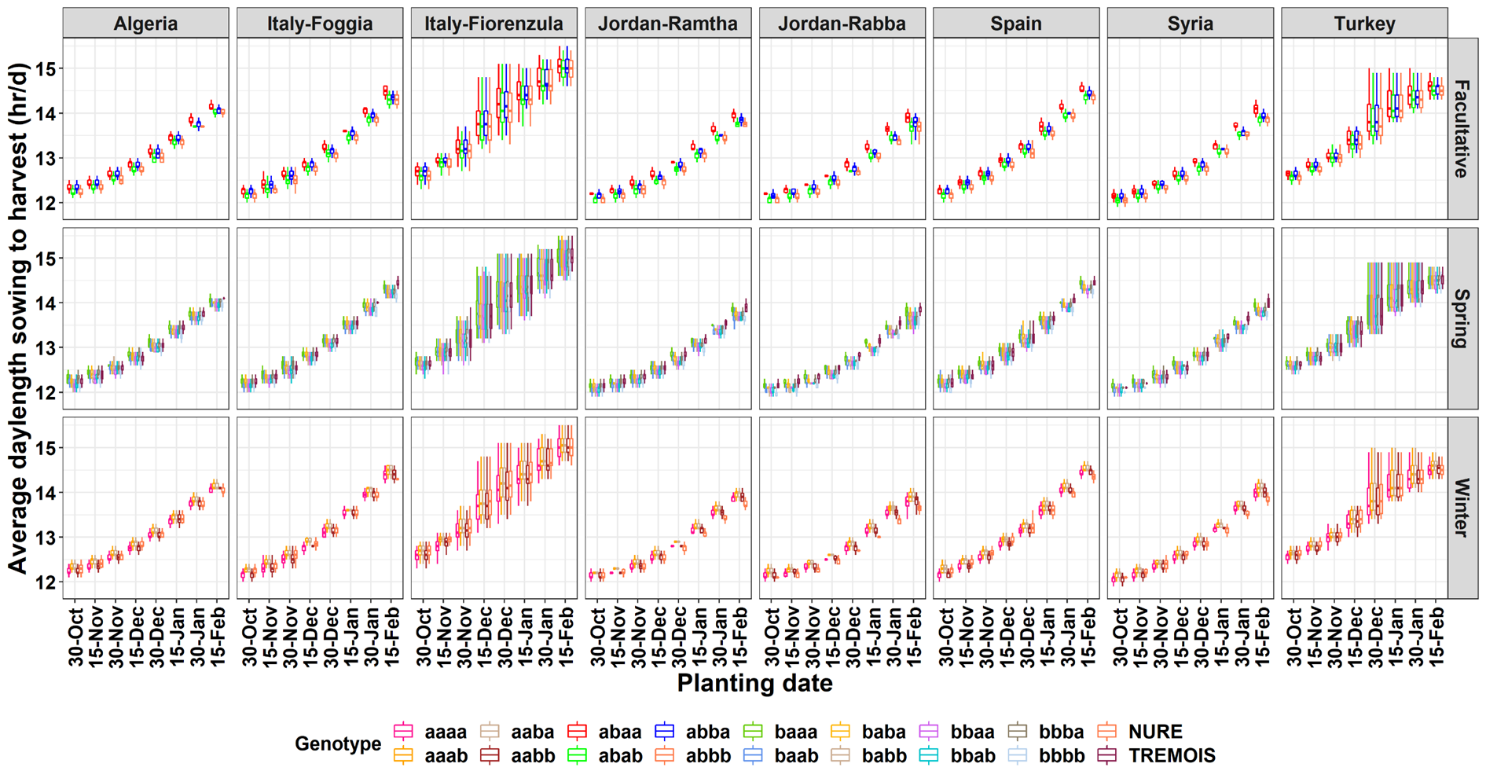


**Supplementary Figure 6.** Simulated values of average daylength from sowing to harvest (hr/d) for each barley type and each planting date. For each boxplot, the end of the horizontal line represents, from the left to the right, the 10^th^ percentile and the 90^th^ percentile. The vertical line of the box, from the left to the right represents the 25^th^ and 75^th^ percentile, respectively.


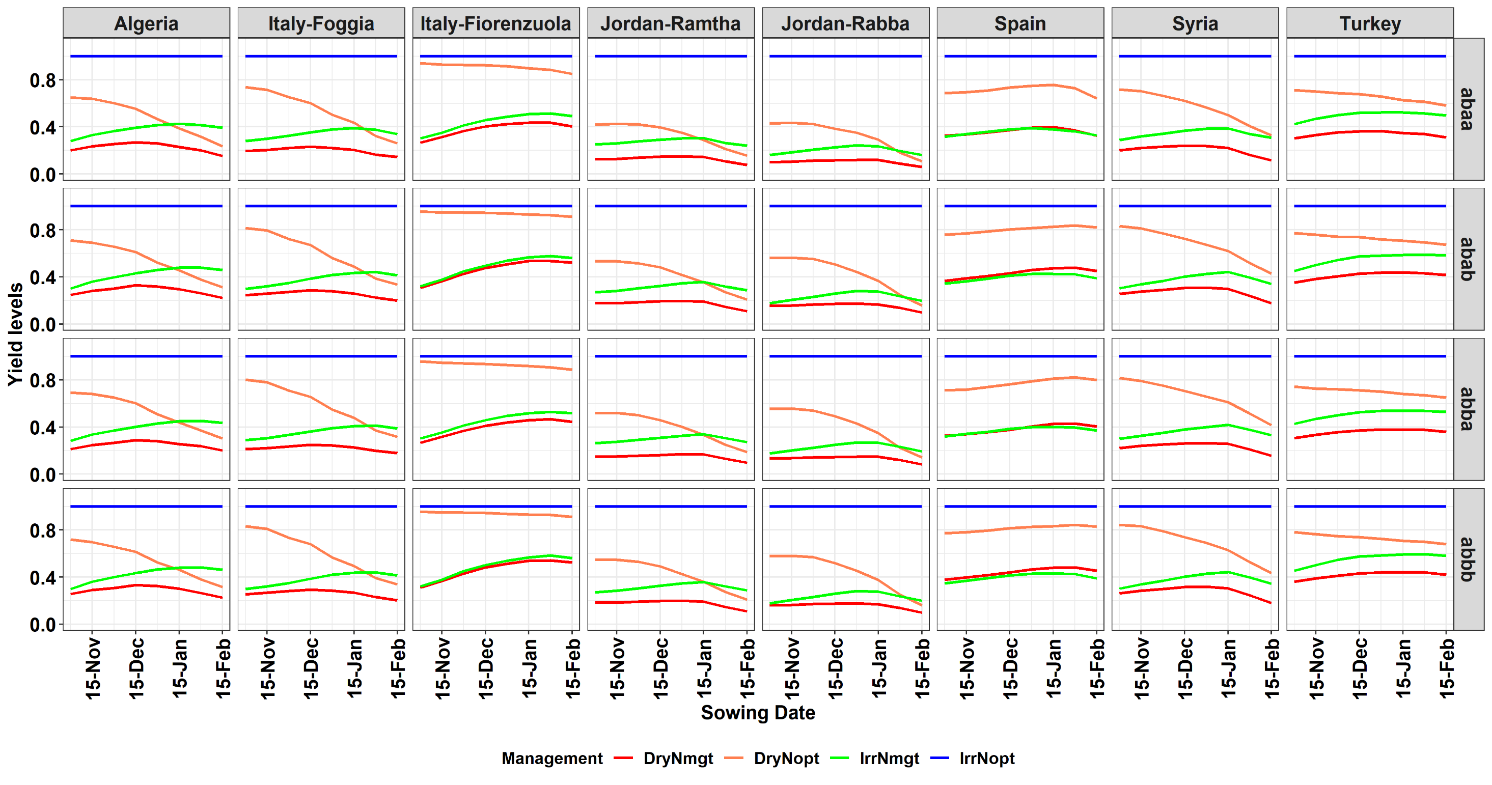


**Supplementary Figure 7.** Yield gap between the Facultative barley type simulated with optimal managements conditions (no water or nitrogen stresses; **IrrNopt** – blue top straight line), no water stress and current nitrogen management (**IrrNmgt** – green line), dryland and optimal nitrogen management (**DryNopt** – orange line), and under current management conditions (**DryNmgt** – red line).


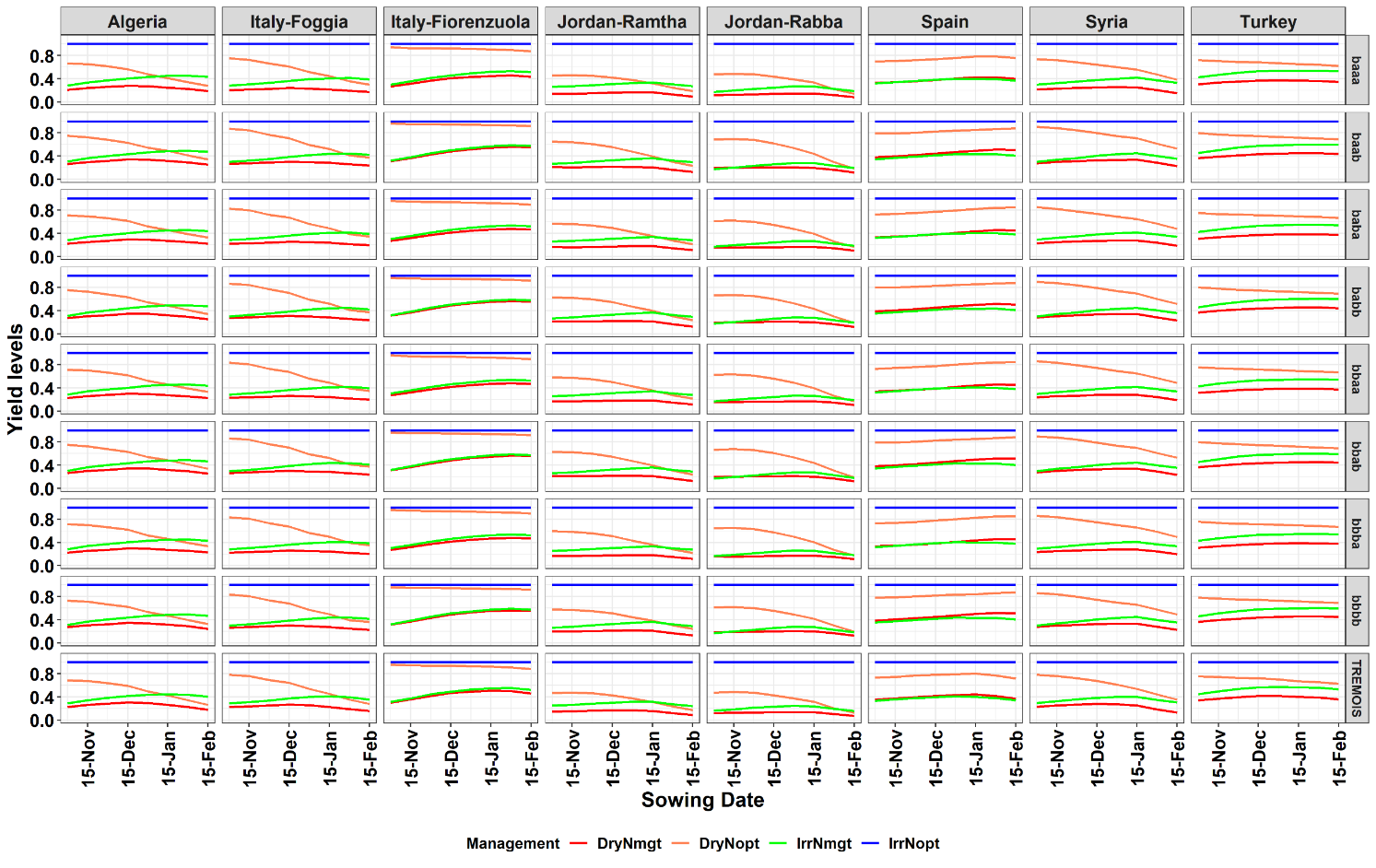


**Supplementary Figure 8.** Yield gap between the Spring barley type simulated with optimal managements conditions (no water or nitrogen stresses; **IrrNopt** – blue top straight line), no water stress and current nitrogen management (**IrrNmgt** – green line), dryland and optimal nitrogen management (**DryNopt** – orange line), and under current management conditions (**DryNmgt** – red line).


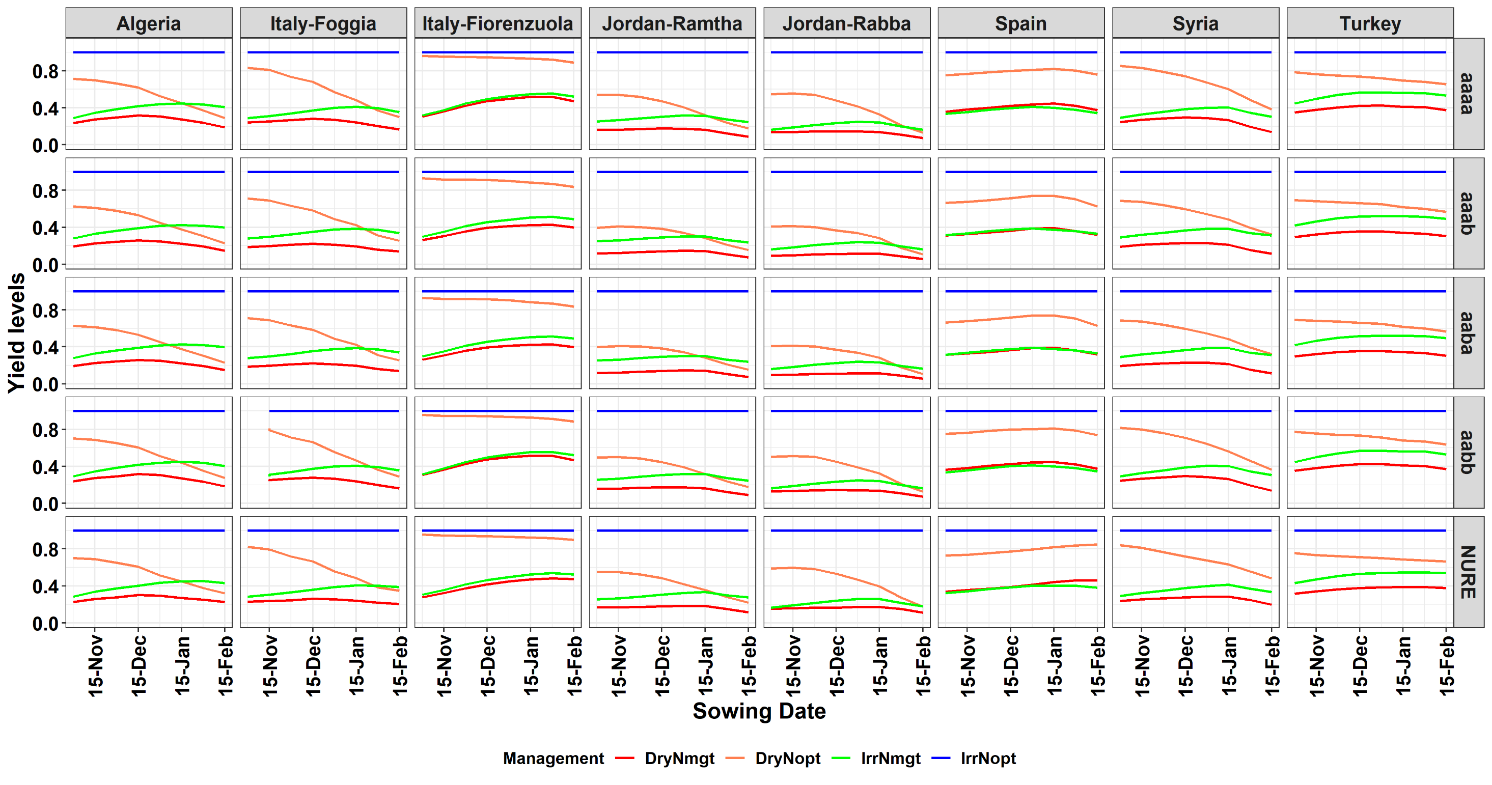


**Supplementary Figure 9.** Yield gap between the Winter barley type simulated with optimal managements conditions (no water or nitrogen stresses; **IrrNopt** – blue top straight line), no water stress and current nitrogen management (**IrrNmgt** – green line), dryland and optimal nitrogen management (**DryNopt** – orange line), and under current management conditions (**DryNmgt** – red line).


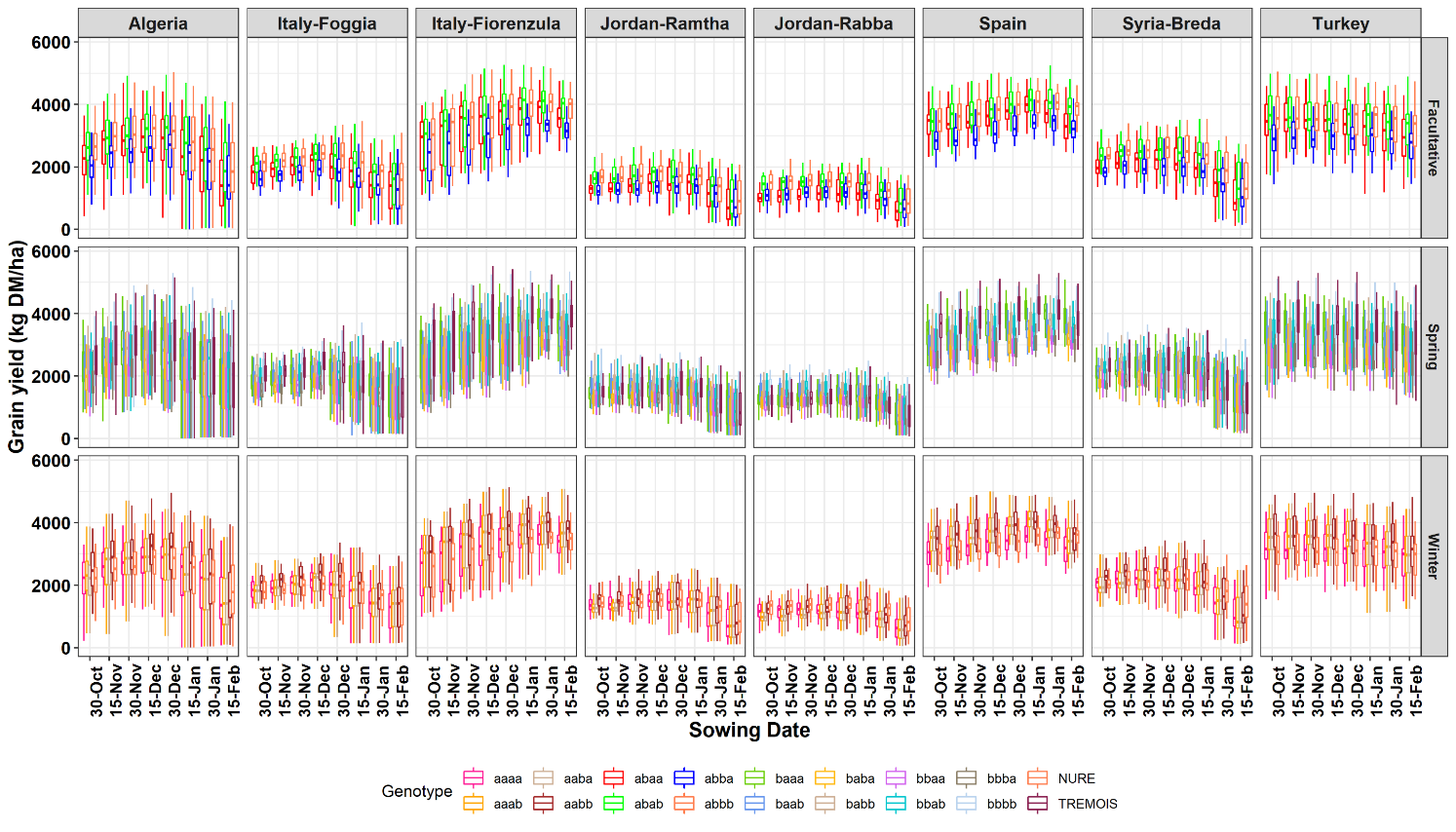


**Supplementary Figure 10.** Simulated crop dry weight for each genetic type and each location for different sowing dates. The rows represent each barely type (from top to bottom, Faculty, Spring, Winter) and the columns each location. For each boxplot, the end of the horizontal line represents, from the left to the right, the 10^th^ percentile and the 90^th^ percentile. The vertical line of the box, from the left to the right represents the 25^th^ and 75^th^ percentile, respectively.

**REFERENCES**

Cammarano, D., Ceccarelli, S., Grando, S., Romagosa, I., Benbelkacem, A., Akar, T., et al. (2019). The impact of climate change on barley yield in the Mediterranean basin. *European Journal of Agronomy* 106**,** 1-11. doi: <https://doi.org/10.1016/j.eja.2019.03.002>.

Francia, E., Tondelli, A., Rizza, F., Badeck, F.W., Li Destri Nicosia, O., Akar, T., et al. (2011). Determinants of barley grain yield in a wide range of Mediterranean environments. *Field Crops Research* 120(1)**,** 169-178. doi: <https://doi.org/10.1016/j.fcr.2010.09.010>.
